# Sensitivity of DECT in ACL tears. A prospective study with arthroscopy as reference method

**DOI:** 10.1177/20584601221075799

**Published:** 2022-03-09

**Authors:** Ann-Sofi Björkman, Håkan Gauffin, Anders Persson, Seppo K. Koskinen

**Affiliations:** 1 Center for Medical Image Science and Visualization (CMIV), 4566 Linköping University, Linköping, Sweden; 2 Department of Radiology in Linköping, and Department of Health, Medicine and Caring Sciences, 123898 Linköping University, Linköping, Sweden; 3 Department of Orthopedics, 4566 Linköping University, Linköping, Sweden; 4 Department of Biomedical and Clinical Sciences, 4566 Linköping University, Linköping, Sweden; 5 Department of Clinical Science, Intervention, and Technology, Division for Radiology, 27106 Karolinska Institute, Stockholm, Sweden

**Keywords:** Knee, ACL, trauma, dual energy CT, ligament tear

## Abstract

**Background:**

CT is often used for fracture evaluation following knee trauma and to diagnose ACL injuries would also be valuable.

**Purpose:**

To investigate the diagnostic accuracy of dual energy CT (DECT) for detection of ACL tears in acute and subacute knee injuries.

**Material and Methods:**

Patients with suspected ACL injury were imaged with DECT and MRI. Clinically blinded DECT images were independently read twice by two radiologists. ACL was classified as normal or abnormal. Arthroscopy served as reference method. Sensitivity and positive predictive value (PPV) were calculated, and diagnostic performance between DECT and MRI was assessed.

**Results:**

48 patients (26 M, 22 F, mean age 23 years, range 15–37 years) were imaged with a mean of 25 days following trauma. Of these, 21 patients underwent arthroscopy with a mean of 195 days after trauma. Arthroscopy revealed 19 ACL tears and 2 ACLs with no tear. The combined sensitivity was 76.3% (95% CI 66.8–85.9) and 86.8 (95% CI 71.9–95.6) for DECT and MRI, respectively. There was no statistically significant difference between these two methods (*p* = .223). The positive predictive value (PPV) was 93.5 (95% CI 84.3–98.2) and 91.7 (95% CI 77.5–98.3) for DECT and MRI, respectively.

**Conclusion:**

DECT has lower sensitivity to detect an ACL rupture than MRI, but the difference is not statistically significant. The PPV is high in both methods.

## Introduction

Anterior cruciate ligament (ACL) injuries are common^1^ and can have major consequences for the individual such as impeding return to sports^2^ and eventually increasing the risk of osteoarthritis.^2,3^ Arthroscopy is the traditional diagnostic method used to identify ACL injuries, and it still remains the reference standard.^4,5^ However, it is an invasive examination with known adverse effects.^6^ MRI is the imaging method of choice for diagnosing ACL injuries, with pooled sensitivity/specificity of 87%/93% for ACL when compared to arthroscopy.^4^ CT, on the other hand, is often used in the emergency department for fracture detection^7^ as well as for preoperative evaluation following knee trauma. In this situation, being able to provide a soft tissue evaluation would be valuable. A previous study using a multi detector computer tomography (MDCT) found that it can detect an intact ACL and posterior cruciate ligament (PCL) with good specificity but that the assessment of torn ligaments was unreliable.^8^ A more recent MDCT study found a sensitivity and specificity of 87.5–100% and 100% respectively for ACL injury when performed in the non-acute phase.^9^ Dual energy CT (DECT) studies on detection of ACL injury are few, done retrospectively, often with MRI as the reference method, and with a limited number of patients (18–27 patients, 6–16 ACL ruptures).^10,11^ The reported sensitivity and specificity for ACL injury range between 78 and 86% and 88 and 100%, respectively. The main objective of this study was to prospectively evaluate the sensitivity of DECT in detecting acute and subacute injuries to the ACL with arthroscopy as reference method.

## Material and methods

The material used in the present study was also used in a previous study^12^ but the data on ACL injury detection has not been published before. In short, this study is part of the prospective NACOX study with the overall aim to evaluate the natural corollaries and recovery after an ACL injury.^13^ Ethical approval from the regional ethical review board (2016/44-31, 2017/221-32 and 2021-00197) and radiation protection committee was obtained. Written informed consent was obtained from all individual participants included in the study. STARD guidelines were followed.^14^

### Patient selection

The inclusion and exclusion criteria were described in the NACOX study protocol.^13^ Briefly, eligibility criteria for the NACOX study were having an ACL injury sustained no more than 6 weeks prior to clinical examination at the orthopedic clinic, being aged 15–40 years at the time of injury, no previous ACL injury/ACL reconstruction on the same knee, no serious concomitant knee injury (e.g., posterior cruciate ligament rupture or a fracture that requires separate treatment) and no other illness or injury that impairs function.

**Figure 1.**
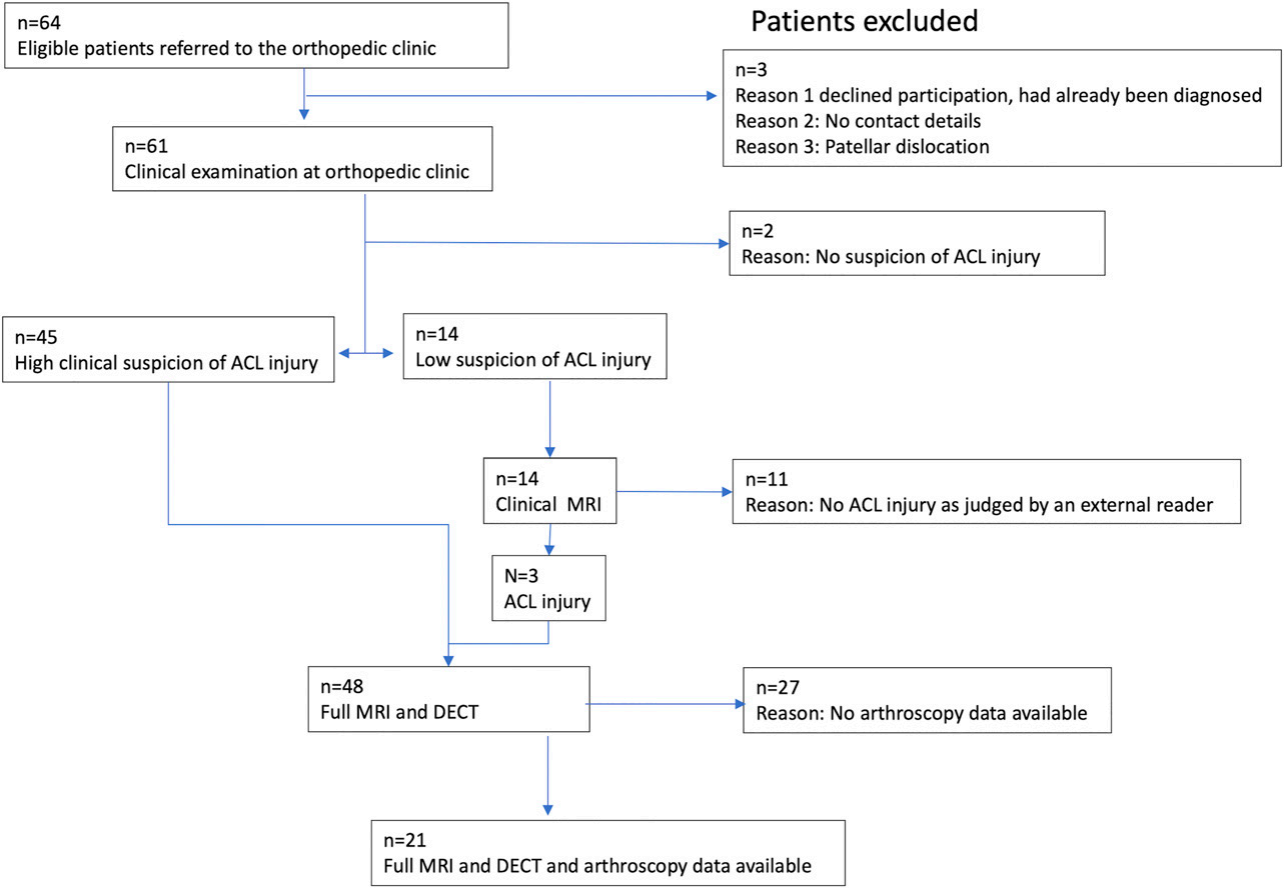
Flowchart depicting patient inclusion as well as reasons for exclusion. Patients with low clinical probability of ACL injury first went through an MRI examination containing only clinical sequences which were interpreted by an external radiologist. Three out of these patients were determined to have an ACL injury and a complete MRI protocol, containing also experimental sequences (intended for other studies), was performed along with DECT. Patients with high clinical suspicion of ACL injury performed the complete MRI protocol and DECT directly. Of these 48 patients, 21 had arthroscopy data and were included in the final study group.

Patients were referred from physiotherapists or the emergency department at the hospital for suspected ACL injury. Clinical examination was performed by an orthopedic surgeon. When suspecting a possible ACL injury, MRI and DECT were performed. All NACOX eligible patients that underwent MRI and DECT from 1 October 2017 to 20 March 2018 were included in the analysis. Of the intended sample size of 50 patients,^15^ we were able to include 48 consecutive patients for imaging. Of these, 21 patients underwent arthroscopy and were used in the final analysis. A flowchart depicting patient inclusion is shown in Figure 1.

### DECT protocol and image post-processing

Patients were scanned bilaterally on a dual source CT scanner (Somatom Definition Force, Siemens Healthcare, Germany). X-ray tubes were set at 80 kV and 150 kV, the latter with a tin filter. The quality reference mAs was 125 mAs with the automatic exposure control turned on (Care Dose). The detector collimation was 128 × 0.6 mm, the gantry rotation 0.5 s, and the pitch 0.7. No contrast agent was used.

Image analysis was done in the Dual Energy CT workflow with the Monoenergetic+ application using a commercial workstation (Syngo.via VB10B_HF05 and VB20A_HF4, Siemens Healthcare, Germany). Radiologists could choose which keV would be simulated from the two datasets obtained as well as freely adjust window settings and do reconstructions in any desired plane.

### MRI protocol

MRI images were acquired bilaterally using a 3.0 T scanner (Ingenia, Philips Medical Systems, Best, The Netherlands) with a 16-channel knee-coil. The protocol was the clinical protocol used at the hospital with an additional 3D sequence (Table 1).

**Table 1.** MRI protocol.

Parameter	Sagittal PD	Axial PD Fat Sat	Sagittal PD FatSat	Coronal PD FatSat	3D PD FatSat
EchoTime, TE (ms)	20	35	30	30	185
Repetition Time, TR (ms)	1800	3981	3400	3572	1300
Echo Train Length, ETL	10	15	15	16	63
Matrix size	516 × 384	332 × 330	468 × 399	516 × 332	228 × 226
Field of View (cm)	160 × 145	140 × 140	160 × 145	160 × 140	144 × 162
Slice Thickness/space (mm)	3/0.3	3/0.3	3/0.3	3/0.3	0.63/-
Scan Time (min)	02:58	04:15	03:56	03:56	06:31

MRI images were analyzed using the hospital’s research PACS (Sectra Workstation, IDS7 19.1, Sectra AB, Linköping, Sweden).

### Image analysis

As DECT is relatively new method to assess the cruciate ligaments, we assessed intra-and interobserver agreement. Therefore, the blinded DECT data of all knees (*n* = 96) was reviewed twice in a randomized fashion with at least 13 days interval (median 65 days) by a radiology resident with 3 years of experience in radiology (AB), and by an experienced radiologist with more than 7 years of experience in musculoskeletal radiology (ML). The same Readers analyzed separately the blinded, randomized MRI data. No clinical data were available during the image analysis. For both DECT and MRI analysis, both knees of each patient were presented together. Two patients had injured their contralateral knee 3 and 5 months earlier and both knees were included. Readers were unaware of which knee had been injured. The presence of ACL injury was noted for each knee separately. ACL was considered normal when fibers could be seen as continuous structures with no signs of discontinuous segments and abnormal when there was discontinuous/complete separation of fibers or avulsion. If the Reader was not confident or consider the ligament unevaluable, it was considered abnormal in the analyses. For the inter- and intraobserver agreement, only data of the injured knee (*n* = 50) were used. Also, consensus MR reading was performed.^12^

### Radiation dose

The CTDIvol as well as the dose-length product were recorded. The effective dose was calculated as described earlier.^16^

### Statistical analysis

The diagnostic performance of DECT and MRI was assessed using arthroscopy data as reference standard. Difference between DECT and MRI was evaluated with Fisher’s exact test. The inter- and intraobserver agreement for ACL injury were determined using the Cohen’s kappa (κ). κ-values were considered as follows: 0.01–0.20 slight agreement, 0.21–0.40 fair agreement, 0.41–0.60 moderate agreement, 0.61–0.80 substantial agreement, and 0.80–0.99 almost perfect agreement.^17^

Statistical analyses were done using a commercial software package SAS/STAT v.9.4 (SAS Institute Inc., Cary, NC, USA).

## Results

### Patients

Twenty-six males and 22 females were recruited with a mean age of 23 years (range 15–37 years). The mean interval between injury and DECT was 25 days (range 4–55 days), and MRI was performed the same day (*n* = 42) or within 7 days (*n* = 6). None of the imaging studies were excluded because of poor technical quality.

Of these 48 patients, 33 patients had partial or complete rupture of the ACL on MR-consensus reading. Arthroscopy data were available for 21 patients (13 males with mean age 22 years, range 16–37, eight females with mean age 22 years, range 15–36). The indication for arthroscopy was knee instability (*n* = 19), meniscal tear (*n* = 1), and limited knee joint range of motion (*n* = 1). The mean interval between injury and DECT/MRI/arthroscopy was 30/31/195 days (range 7–55/15-55/47–349 days), respectively. The respective median time was 26/26/168 days.

### ACL injury

**Figure 2.**
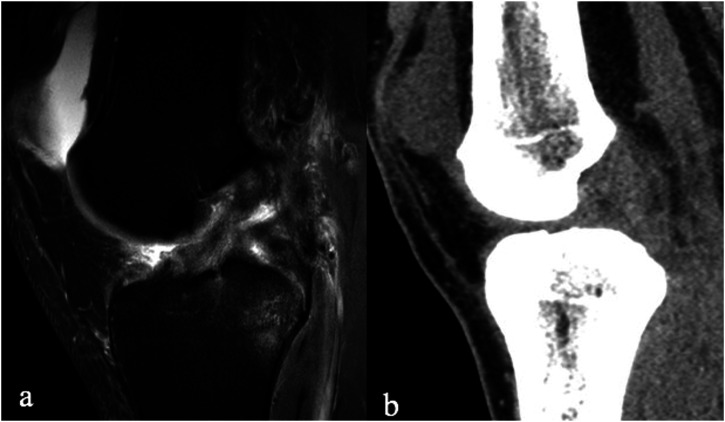
True positive. A 24-year-old male with an arthroscopy verified torn ACL in both imaging modalities. Sagittal MRI (PD, TE 20 ms., TR 1800 ms., slice thickness 3 mm) (a) and sagittal DECT (b). The Readers were able to freely adjust window settings and do reconstructions in any desired plane when analyzing DECT images.

**Figure 3.**
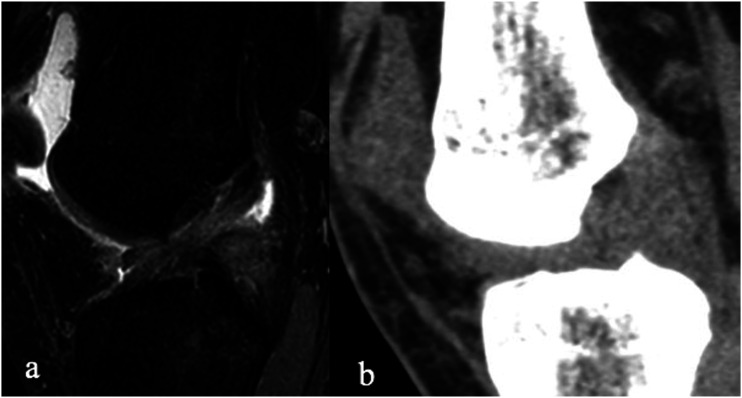
False positive: A 27-year-old male with a normal ACL in arthroscopy that was classified as torn in MRI (Sagittal PD fat sat TE 30 ms., TR 3400 ms., slice thickness 3 mm) (a) and in DECT (b). The Readers were able to freely adjust window settings and do reconstructions in any desired plane when analyzing DECT images.

Out of 21 patients, 19 had partial or complete rupture of the ACL and two intact ACL on arthroscopy. The combined sensitivity was 76.3% (95% CI 66.8–85.9) and 86.8 (95% CI 71.9–95.6) for DECT and MRI, respectively. There was no statistically significant difference between these two methods (*p* = .223). The corresponding positive predictive values for DECT and MRI were 93.6% (95% CI 84.3–98.2) and 91.67 (77.5–98.2) (n.s.), respectively. Due to low number of negative cases (*n* = 2), the specificity and negative predictive values were not calculated. The results for separate reading are presented in Table 2, and an example of a true and false positive is shown in Figures 2 and 3.

**Table 2.** The sensitivity and positive predictive value (PPV) of DECT and MRI in detecting an ACL tear.

	Sensitivity	PPV
DECT combined	76.30 (66.76–85.87)	93.55 (84.30–98.21)
MRI combined	86.84 (71.91–95.59)	91.67 (77.53–98.25)
DECT R1, first reading	57.89 (33.50–79.75)	91.67 (61.52–99.79)
DECT R1, second reading	78.95 (54.43–93.95)	93.75 (69.77–99.84)
DECT R2, first reading	84.21 (60.42–96.62)	94.12 (71.31–99.85)
DECT R2, second reading	84.21 (60.42–96.62)	94.12 (71.31–99.85)
MRI R1	89.47 (66.86–98.70)	89.47 (66.86–98.70)
MRI R2	84.21 (60.42–96.62)	94.12 (71.31–99.85)

R1: Reader 1, R2: Reader 2.

For DECT, the intraobserver agreement was moderate (κ 0.41; 95% CI 0.17–0.65) for Reader 1 and substantial (κ 0.70; 95% CI 0.48–0.91) for Reader 2. Interobserver agreement was fair (κ 0.39; 95% CI 0.20–0.59). For MRI, the interobserver agreement was almost perfect (κ 0.87; 95% CI 0.74–1.00)

### Radiation

The mean CTDIvol was 4.0 mGy. The mean dose-length product (DLP) was 87.8 mGycm. Using the factor k=0.0004 as described earlier^16^ the mean effective dose was 0.04 mSv (range 0.030–0.045 mSv, SD 0.003 mSv).

## Discussion

In this prospective study we wanted to investigate the diagnostic accuracy of DECT for detection of ACL tears in the acute and subacute knee injuries with arthroscopy as the reference standard. The combined sensitivity and positive predictive value for DECT were 76.3% and 93.6%, respectively. In contrast, the corresponding values for MRI were 86.8% and 91.7%. This difference was not, however, statistically significant.

Previous studies on MDCT or DECT in assessing ACL have mainly had MRI as the reference standard. For example, in a study using a four-slice MDCT the sensitivity was 58% for detection of ACL injury^8^ while a more recent study using a 64 slice MDCT found a sensitivity of 87.5–100%.^9^ DECT studies have found sensitivity levels between 78 and 86%.^10,11^ A case–control study of 16 ACL rupture patients, with MRI, arthroscopy (*n* = 14) and clinical data as the reference standard, demonstrated DECT to have good sensitivity (50–100%) and interobserver agreement for the detection of complete ACL tears.^11^ However, the mean time interval between MRI and DECT was 39 days and mean time from injury to DECT was 59 days, that is, almost twice as long as in our study. The lower sensitivity of the four-slice MDCT study^8^ is probably mostly attributable to technical limitations of the CT at the time compared to more recent studies with higher resolution.^9–11^ Our results are in keeping with the more recent results except for the recent MDCT study demonstrating a higher sensitivity.^9^ This might be attributable to the time delay from referral to imaging which was 132 days,^9^ hence representing a chronic phase compared to the mean 31 days from trauma to imaging in our study. After a traumatic injury, there are often joint effusion and edematous changes in the joint which can interfere with the evaluation of ACL integrity. This might also, at least partly, explain the difference in our result compared to those reported previously.^11^ In a healthy knee, the ACL is usually well delineated and surrounded by fat increasing the confidence of excluding a torn ACL.

We did not use the material decomposition technique for tendon specific color display available in the workstation since studies have shown it to be inferior to normal gray scale visualization for ACL detection.^10,11^

ACL injury intraobserver agreement was moderate to substantial, and lower than the substantial to almost perfect agreement (κ= 0.85 and 0.72) presented in a previous DECT study.^10^ Interobserver agreement was fair and less than in previous studies (κ=0.85–1.00).^9,10^ Besides reader experience, this might be explained by the number of cases reviewed; the large number of knees as in the present study compared to the previously mentioned studies (*n* = 40 and 18, respectively^9,10^) gives less time for each evaluation but provides a higher degree of statistical certainty. Also, the retrospective design of one study^10^ gives a possible selection bias as only patients that had undergone both DECT and MRI were imaged. Finally, acute traumatic changes might make diagnostics more difficult compared to imaging an injured knee in the chronic phase.^9^

The radiation dose was very low, the mean CTDIvol was 4.0 mGy and the estimated effective dose was 0.04 mSv. This is much less than the 0.24 mSv of a previous DECT study on ACL.^10^ This means that a modern CT machine provides low radiation doses, making the dose and radiation risk almost negligible when examining the knee and peripheral joints.

The strength of the present study is the prospective design and relatively short time difference between DECT and MRI examinations meaning bias of status change between examinations is unlikely. Further, the mixed experience of the reading radiologists showed that even unexperienced readers can perform rather well; hence, the method is easy to adopt.

Because the NACOX study aims at studying the natural corollaries after an ACL injury, the age of subjects was limited to 15–40 years which limits the ability to generalize our results to older populations, but ACL-surgery is often performed in a young population and thus most important to find in this age-group.

The limitation of the current study is the low number of true negatives. In Sweden only about half of the ACL-injured patients undergo an ACL-reconstruction. Also, the mean time from injury to operation is about 500 days.^18^ On the other hand, an unstable meniscal injury could be a reason for an early arthroscopy, but more than 40% of ACL-injured athletes do not have an associated meniscal tear.^19^ Moreover, a complete healing can be seen in over 50% of meniscal tears left in situ during anterior cruciate ligament reconstruction.^20^ These facts could explain that only 21 of 33 (64%) patients underwent arthroscopy after the ACL injury had been diagnosed by MRI.

Both knees were presented together when evaluating the images which might have facilitated ACL detection considering that it is unlikely to have bilateral ACL disruptions. On the other hand, in clinical practice, both knees are usually included in the field of view even though not all institutions present them to the radiologist as in our case. Also, our study has not addressed whether the use of dual energy adds value compared to a modern conventional MDCT. The obvious advantage of DECT over conventional MDCT would be the bone marrow lesion visualization which has been shown to correlate rather well with MRI^12, 21–23^ and serving as a tell-tale sign of a possible ACL injury.^24^

Further research addressing how well DECT performs in the injured knee using the combination of typical bone contusion pattern, fractures and ACL injury would be of value. DECT will not replace MRI for detection of ACL tears but there are, however, several other clinical benefits: In preoperative planning for fracture surgery a CT is often performed and being able to exclude an ACL injury would be an important information. Also, preoperative planning is usually done during the first days following trauma while our study had a mean 31 days from trauma to imaging further limiting the direct implication of our results. Another clinical usage would be for patients with external fixation. The MR-compatible frame may allow the MR examination, but the metal artifacts impair MRI image quality, making the ACL difficult or even impossible to assess. Furthermore, in patients with claustrophobia or MR incompatible or contraindicated metal implants, DECT may be an alternative imaging method. CT arthrography could be an option, but it is an invasive examination, and may not be commonly available.

In conclusion, DECT has lower sensitivity to detect an ACL tear than MRI, but the difference is not statistically significant. The PPV is high in both methods.

## References

[1] Montalvo AM Schneider DK Yut L , et al. “What’s my risk of sustaining an ACL injury while playing sports?” A systematic review with meta-analysis. Br J Sports Med 2019; 53: 1003–1012. 29514822 10.1136/bjsports-2016-096274 PMC6561829

[2] Ajuied A Wong F Smith C , et al. Anterior cruciate ligament injury and radiologic progression of knee osteoarthritis: a systematic review and meta-analysis. Am J Sports Med 2014; 42: 2242–2252. 24214929 10.1177/0363546513508376

[3] Snoeker B Turkiewicz A Magnusson K , et al. Risk of knee osteoarthritis after different types of knee injuries in young adults: a population-based cohort study. Br J Sports Med 2020; 54: 725–730. DOI: 10.1136/bjsports-2019-100959. 31826861

[4] Phelan N Rowland P Galvin R , et al. A systematic review and meta-analysis of the diagnostic accuracy of MRI for suspected ACL and meniscal tears of the knee. Knee Surg Sports Traumatol Arthrosc 2016; 24: 1525–1539. 26614425 10.1007/s00167-015-3861-8

[5] Bari AA Kashikar SV Lakhkar BN , et al. Evaluation of MRI versus arthroscopy in anterior cruciate ligament and meniscal injuries. J Clin Diagn Res 2014; 8: RC14–RC18. 25654007 10.7860/JCDR/2014/10980.5331 PMC4316313

[6] Hetsroni I Lyman S Do H , et al. Symptomatic pulmonary embolism after outpatient arthroscopic procedures of the knee: the incidence and risk factors in 418,323 arthroscopies. J Bone Joint Surg Br 2011; 93: 47–51. 21196542 10.1302/0301-620X.93B1.25498

[7] Mustonen AOT Koskinen SK Kiuru MJ . Acute knee trauma: analysis of multidetector computed tomography findings and comparison with conventional radiography. Acta Radiol 2005; 46: 866–874. 16392612 10.1080/02841850500335135

[8] Mustonen AOT Koivikko MP Haapamaki vv , et al. Multidetector computed tomography in acute knee injuries: assessment of cruciate ligaments with magnetic resonance imaging correlation. Acta Radiol 2007; 48: 104–111. 17325934 10.1080/02841850601045138

[9] Heffernan EJ Moran DE Gerstenmaier JF , et al. Accuracy of 64-section MDCT in the diagnosis of cruciate ligament tears. Clin Radiol 2017; 72: 611.e1–611.e8. 10.1016/j.crad.2017.01.006 28214478

[10] Peltola EK Koskinen SK . Dual-energy computed tomography of cruciate ligament injuries in acute knee trauma. Skeletal Radiol 2015; 44: 1295–1301. 26025120 10.1007/s00256-015-2173-x

[11] Glazebrook KN Brewerton LJ Leng S , et al. Case-control study to estimate the performance of dual-energy computed tomography for anterior cruciate ligament tears in patients with history of knee trauma. Skeletal Radiol 2014; 43: 297–305. 24337491 10.1007/s00256-013-1784-3

[12] Björkman A-S Koskinen SK Lindblom M , et al. Diagnostic accuracy of dual-energy CT for detection of bone marrow lesions in the subacutely injured knee with MRI as reference method. Acta Radiol 2020; 61: 749–759. 31581782 10.1177/0284185119877343

[13] Kvist J Gauffin H Tigerstrand Grevnerts H , et al. Natural corollaries and recovery after acute ACL injury: the NACOX cohort study protocol. BMJ Open 2018; 8. Available at: http:\\bmjopen.bmj.com/content/8/6/e020543.abstract. 10.1136/bmjopen-2017-020543 PMC6020951 29950463

[14] Cohen JF Korevaar DA Altman DG , et al. STARD 2015 guidelines for reporting diagnostic accuracy studies: explanation and elaboration. BMJ Open 2016; 6: e012799. 10.1136/bmjopen-2016-012799 PMC5128957 28137831

[15] Whitehead AL Julious SA Cooper CL , et al. Estimating the sample size for a pilot randomised trial to minimise the overall trial sample size for the external pilot and main trial for a continuous outcome variable. Stat Methods Med Res 2016; 25: 1057–1073. 26092476 10.1177/0962280215588241 PMC4876429

[16] Saltybaeva N Jafari ME Hupfer M , et al. Estimates of effective dose for CT scans of the lower extremities. Radiology 2014; 273: 153–159. 24937693 10.1148/radiol.14132903

[17] Landis JR Koch GG . The measurement of observer agreement for categorical data. Biometrics 1977; 33: 159–174. 843571

[18] The Swedish knee ligament registry . Annual report 2020. www.aclregister.nu.

[19] Binfield PM Maffulli N King JB . Patterns of meniscal tears associated with anterior cruciate ligament lesions in athletes. Injury 1993; 24: 557–561. 8244553 10.1016/0020-1383(93)90038-8

[20] Pujol N Beaufils P . Healing results of meniscal tears left in situ during anterior cruciate ligament reconstruction: a review of clinical studies. Knee Surg Sports Traumatol Arthrosc 2009; 17: 396–401. 19183957 10.1007/s00167-008-0711-y

[21] Cao JX Wang YM Kong XQ , et al. Good interrater reliability of a new grading system in detecting traumatic bone marrow lesions in the knee by dual energy CT virtual non-calcium images. Eur J Radiol 2015; 84: 1109–1115. 25816992 10.1016/j.ejrad.2015.03.003

[22] Seo SH Sohn YJ Lee CH , et al. Dual-Energy CT for detection of traumatic bone bruises in the knee joint. J Korean Soc Radiol 2013; 69: 487–494.

[23] Pache G Krauss B Strohm P , et al. Dual-Energy CT Virtual Noncalcium Technique: Detecting Posttraumatic Bone Marrow Lesions—Feasibility Study. Radiology 2010; 256: 617–624. 20551186 10.1148/radiol.10091230

[24] Lee CH Tan CF Kim O , et al. Osseous injury associated with ligamentous tear of the knee. Can Assoc Radiol J 2016; 67: 379–386. 27499452 10.1016/j.carj.2016.02.002

